# Microbiome Flora and Metabolomics Analysis of Mung Bean Sour Liquid in Luoyang, China

**DOI:** 10.3390/foods14030511

**Published:** 2025-02-05

**Authors:** Xinyi Zhang, Shengjuan Zhao, Jiangfeng Yuan, Lixing Feng

**Affiliations:** 1 College of Food and Bioengineering, Henan University of Science and Technology, Luoyang 471023, China; xinyizhang2022@126.com; 2 Henan International Joint Laboratory for Food Green Processing and Quality Safety Control, Luoyang 471023, China; 3 Shanghai Majorbio Bio-Pharm Technology Co., Ltd., Shanghai 200120, China; fenglixing2000@163.com

**Keywords:** mung bean sour liquid, microbiome flora, volatiles, metabolites, non-target LC-MS

## Abstract

In order to reveal the fermentation microflora and fermentation metabolites of traditional mung bean sour liquid (MBSL) in Luoyang area, China, this experiment was sampled from four administrative districts of Luoyang, and volatile metabolites and non-targeted metabolites were detected and analyzed by HS-SPME-GC-MS and LC-MS, and bacterial and fungal sequencing were analyzed by Illumina MiSeq high-throughput sequencing technology. And the correlation between microorganisms and metabolites was conducted. The results showed that 42 volatiles were detected in four MBSL samples named Jianxi (JX), Liujia (Liu), LiJia (LJ), Majia (MJ), with 11 identical volatile flavor subtances, and the highest content of esters was found in JX, Liu, and LJ, and the highest content of acids was found in MJ. A total of 1703 non-targeted metabolites were identified, and there were more types of amino acids, carbohydrates, fatty acids and their complexes, flavonoids, carbonyl compounds, and organic acids, accounting for 40.93%. High-throughput sequencing results showed that there were nine bacterial and fungal genera with an average abundance of more than 1%, and the dominant genera mainly belonged to lactic acid bacteria and yeast. The composition of dominant genera was different in different workshop samples, and the abundance of fungal genera differed greatly. Among the volatile substances, Methyleugenol, a volatile component, was related to more bacteria, and ketones and hydrocarbons may be more closely associated with bacteria. Acetic acid and Oxalic acid may be more closely related to fungi, while some esters were more closely related to both fungal and bacterial genera. For non-target metabolites, amino acid and alcohol metabolites may be more influenced by bacteria, and organic acids and flavonoids may be more influenced by fungi.

## 1. Introduction

Sour liquid noodles are a popular traditional snack in Luoyang, loved for their unique sour taste, rich nutrition, and suitability for all ages. There is a saying in Luoyang: “If you heat the sour liquid noodles three times, you do not desire meat”. The main ingredient is noodles, but the key component is the broth, which is not made with regular water but rather with a fermented mung bean sour liquid (MBSL).

MBSL is made by soaking mung beans in water for about 12 h until they expand, then adding about 15 to 20 times the amount of water and grinding them into a coarse paste using a stone mill or beater. The liquid is filtered through a 120-mesh sieve and gauze to remove the residue and then placed in a bowl or jar for fermentation. Once the liquid becomes sour, it is ready to be used for cooking noodles [1]. This production technique has been recognized as part of Luoyang’s intangible cultural heritage.

Mung beans, as the raw material for MBSL, are rich in nutrients and have high food value, making them particularly popular in Asian countries [2]. It is reported that mung beans contain 22.1 g/100 g protein (1–3 times higher than cereal crops), with the protein mainly consisting of globulins. The amino acid composition is complete and well balanced, containing methionine, tryptophan, lysine, leucine, and threonine, with the highest content of phenylalanine and lysine. In addition, mung beans contain many bioactive compounds, such as flavonoids, polysaccharides, polypeptides, coumarins, and alkaloids [3,4,5]. The fermented MBSL also contains abundant proteins, amino acids, organic acids, minerals (such as Ca, Mg, Zn, Fe, Cu), dietary fibers, and other nutrients. These components contribute to the unique flavor of MBSL as well as its health benefits, such as stimulating appetite, promoting digestion, cooling the body, diuretic effects, and relieving cough [6].

With the growing pursuit of health, MBSL has gained increasing attention. It is easy to digest and can stimulate appetite, making it especially popular in Luoyang, particularly among people living in the old town district, Luoyang. Locals often go to buy the sour liquid early in the morning, so that they can enjoy it for lunch, and many local restaurants also serve this snack. Currently, MBSL is not only used for cooking noodles but also serves as a base broth for hotpot. There are now some sour soup hotpot restaurants in Luoyang. MBSL has attracted the attention of Chinese nutrition and health experts, and there is even a demand for its drying and development into convenience foods.

MBSL is produced through microbial fermentation. Li et al. [1] reported the volatile metabolites and microbial communities of MBSL in Luoyang. Their findings showed that *Lactobacillus*, *Algoriella*, *Leuconostoc*, and *Lelliottia* were the main microorganisms. Fan et al. [7] performed bacterial 16S rDNA sequencing on MBSL samples from six Luoyang workshops and found that the dominant bacterial genus was *Lactobacillus*, and functional predictions indicated that the unique sour taste of mung bean sour paste is related to the amino acid metabolism and lactic acid synthesis of these bacteria.

At present, many MBSL workshops in Luoyang have been inherited for decades, distributed in various administrative districts, and they have formed the unique taste and quality of MBSL. But fermentation metabolites and their metabolic pathways are not yet fully understood. Moreover, there are still problems such as workshop-type small-scale production, backward technology, greater dependence on the natural environment and artificiality, unstable product quality, lack of industrial development, low utilization of resources and food safety, etc., with no industrial scale production and no in-depth theoretical research to support its development.

In the experiment, microbial communities and metabolites of MBSL samples were comprehensively analyzed by aseptically sampling from four pulp houses in different administrative districts of Luoyang. The microbial community was determined by high-throughput sequencing, and the fermentation metabolites were determined by HS-SPME/GC-MS and LC-MS, and the correlation between metabolites and microorganisms was evaluated, so as to analyze the constituents of the microorganisms and metabolites of MBSL in the Luoyang area, and to find out the core functional microorganisms, in order to provide a certain theoretical basis for the production and quality control of MBSL.

## 2. Materials and Methods

### 2.1. Sample Collection

Aseptically take three samples from each of the four workshops in December, grouped separately as Jianxi (JX), Liujia (Liu), LiJia (LJ), Majia (MJ), and the distribution of the specific locations is shown in Table 1. After the samples were collected, they were transported at 4 °C to the laboratory. And then samples were aseptically divided into 10-mL sterile centrifuge tubes and placed at −80 °C to be used for microbiological analysis and metabolite analysis within a week.

### 2.2. Volatile Substances Detection by HS-SPME-GC-MS

The volatile metabolites in MBSL Samples were tested using an Agilent 8890-7000D gas chromatography-mass spectrometer (GC-MS, Agilent, Palo Alto, CA, USA). Sample pretreatment: 4 mL MBSL samples were placed into a 20-mL headspace vial, the internal standard (n-pentadecane-d32 50 g/mL) 5.0 µL was added, and then the headspace vial was immediately sealed. Solid Phase Micro-Extraction (SPME): equilibrium at 80 °C for 20 min, adsorption at 80 °C for 20 min using a 50/30 μ m divinylbenzene/carbene/polydimethylsiloxane extraction head, and desorption at 250 °C for 2 min.

Chromatographic conditions: The volatile compounds were separated by an Agilent VF-WAXms capillary column (25 m × 0.25 mm × 0.2 µm). The injection port temperature was 240 °C, the carrier gas was high-purity helium gas at a 1.5 mL/min flow rate. The heating program was as follows: initial temperature 40 °C, rising to 150 °C at 15 °C/min, then rising to 230 °C at 5 °C/min, and then 230 °C for 5 min.

Mass spectrometry conditions: The electron ionization (EI) was set at 70 eV, ion source temperature was 230 °C, quadrupole temperature was 150 °C. The scanning mode was full scan mode (SCAN), with a quality scanning range of *m*/*z* 50–500 and a scanning frequency of 3.2 scan/s.

The raw data were preprocessed using MassHunter workstation Quantitative Analysis (v10.0.707.0) software for peak extraction, alignment, and other data preprocessing operations. Metabolites were annotated using NIST, Fiehn, MS-DIAL public databases, and self-built databases to obtain metabolite identification results and data matrices. Metabolites were identified using mass spectrometry (MS) matching scores. Next, the identity of metabolites can be further confirmed by calculating the retention index of each component and comparing it with the retention index in the database, and metabolites with RSD greater than 30% are removed.

Relative quantitation: If the concentration of the volatile metabolite is C, the peak area of the volatile metabolite is S, the peak area of the internal standard is S_0_, and the internal standard concentration is C_0_, then the concentration of the volatile metabolite can be estimated according to the formula C = S/S_0_ × C_0._ The relative content refers to the percentage of the concentration of a substance to all substances.

### 2.3. Non-Target LC-MS Detection and Analysis

A sample of 200 µL was accurately transferred to a 1.5-mL centrifuge tube, and 800 µL extraction solution (methanol: acetonitrile = 1:4 (*v*/*v*)) was added. Vortex mixing for 30 s, 4 °C ultrasonic extracting for 30 min, and standing at −20 °C for 30 min were followed by centrifugation at 4 °C 13,000 rpm for 15 min. Then, the supernatant was removed and dried with nitrogen gas. After that, 120 μL acetonitrile aqueous solution (*v*/*v* = 1:1) was added for redissolution, the vortex was mixed for 30 s, the ultrasonic was extracted at 4 °C for 5 min, and it was centrifuged for 15 min at 4 °C 13,000 rpm. Finally, 100 µL supernatant was transferred to the injection vial for analysis.

A total of 3 μL supernatant was separated and analyzed by using Thermo Fisher Scientific’s UHPLC-Q Exactive HF-X Mass spectrometer. Liquid chromatography-mass spectrometry conditions: Acquity UPLC HSST3 column (100 mm × 2.1 mm i.d., 1.8 µm; Waters, Milford, CT, USA). Mobile phase A was 95% water + 5% acetonitrile (containing 0.1% formic acid), and mobile phase B was 47.5% acetonitrile + 47.5% isopropanol + 5% water (containing 0.1% formic acid). The column temperature was 40 °C. The gradient used was: 0–20% (B), 0–3 min; 20–35% (B), 3–4.5 min; 35–100% (B), 4.5–5 min; 100% (B), 5–6.3 min; 0% (B), 6.3–8 min. The flow rate was 0.4 mL/min.

The sample was ionized by electric spray, and the mass spectrum signal was collected by positive and negative ion scanning modes, respectively. The scanning range was 70–1050 *m*/*z*, the sheath gas flow rate was 50 psi, the auxiliary gas flow rate was 13 psi, the auxiliary gas heater temperature was 425 °C, the ion spray voltage floating (ISVF) was −3500 V in negative mode and +3500 V in positive mode, normalized collision energy, MS/MS rolling at 20–40–60 V [8].

The raw data were imported into the metabolomics processing software Progenesis QI v3.0 (Waters Corporation, Milford, CT, USA) for baseline filtering, peak identification, retention time correction, peak alignment, etc., and the main databases HMDB (https://hmdb.ca/, accessed on 24 January 2024), Metlin (https://metlin.scripps.edu/, accessed on 24 January 2024), and other mainstream public databases, as well as self-built databases, were used to identify metabolites by characteristic peak searching. The MS and MS/MS mass spectrometry information was matched with the metabolic database, the mass error of MS was set to less than 10 ppm, and metabolites were identified based on the secondary mass spectrometry matching score. During data processing, metabolites with an RSD greater than 30% were removed.

### 2.4. Microbial Community Analysis

After genomic DNA extraction of the MBSL samples was completed according to the instructions, the DNA purity and concentration were checked by NanoDrop2000 (Thermo Fisher Scientific, Waltham, MA, USA). The polymerase chain reaction (PCR) amplification of the bacterial and fungal genes was, respectively, performed with the universal primer pairs 338F/608R and ITS1F/ITS2R [9,10]. The amplicon products were judged by 2% agarose gel electrophoresis. The PCR products were purified using the AxyPrep DNA Gel Extraction Kit (Axygen, Tewksbury, CA, USA), and high-throughput sequencing was performed using Illumina MiSeq PE300 platform at Shanghai Majorbio Bio-Pharm Technology Co., Ltd. (Shanghai, China).

According to SILVA (https://www.arb-silva.de/) and UNITE (https://ngdc.cncb.ac.cn/databasecommons/database/id/4075, accessed on 24 January 2024), the Qiime platform (http://qiime.org/scripts/assign_taxonomy.html, accessed on 24 January 2024) and RDP classifier Bayesian algorithm (version 2.11 http://sourceforge.net/projects/rdp-classifier/, accessed on 24 January 2024) were used to classify bacterial and fungal OUT representative sequences with 97% similarity level, respectively.

### 2.5. Statistical Analysis

Three repeated analyses were conducted on MBSL samples, and the results were all expressed as the average of three replicates. Based on the Bray–Curtis distance, a principal component analysis (PCA) and orthogonal partial least squares discriminant analysis (OPLS-DA) were performed on volatile metabolites using SIMCA software (Version 14.1), and significant differences in metabolites were determined by combining VIP values with significance tests (*p* < 0.05). The heatmaps were drawn using TBtools software (v2.136). Metabolic enrichment and pathway analysis were performed according to the KEGG database (https://www.genome.jp/kegg/, accessed on 24 January 2024). The correlation analysis between microorganisms and metabolites were based on the Spearman correlation coefficient, and the relevant network diagram was drawn by Cytoscape software (v3.10.2).

## 3. Results and Analysis

### 3.1. Sensory Description of MBSL Samples

There were certain differences in the sensory characteristics of the four MBSL samples, as described in Table 2.

### 3.2. Detection and Analysis of Volatile Metabolites in MBSL

#### 3.2.1. Analysis of Volatile Metabolites Composition and Content

**Figure 1:**
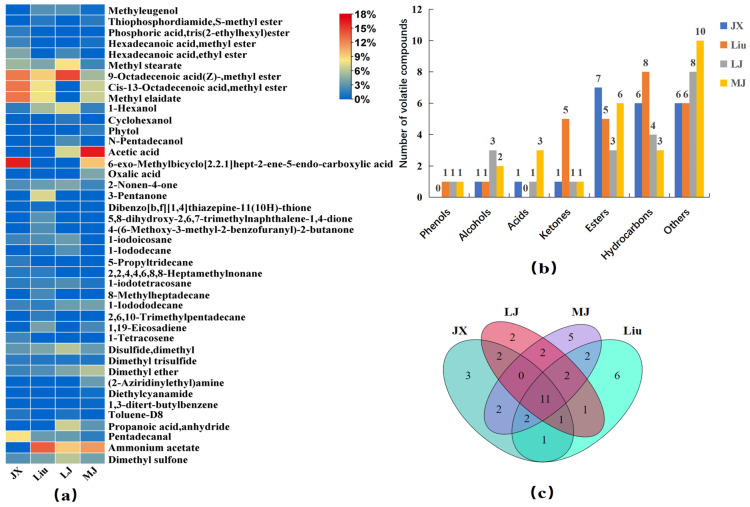
The contents and numbers of volatile compounds in MBSL samples. Notes: (**a**) heatmap of percent content, (**b**) the number of different volatile substances, (**c**) Venn diagram of volatile metabolites.

HS-SPME-GC-MS was used to analyze the volatile metabolites in the four MBSL samples, and a total of 42 volatiles were detected (Table S1), including 1 phenol, 4 alcohols, 3 acids, 5 ketones, 8 esters, 10 hydrocarbons, and 11 other compounds. As shown in Figure 1a,b, 23, 26, 21, and 26 volatile metabolites were detected in JX, Liu, LJ, and MJ, respectively. The highest content of acids was found in MJ, accounting for 30.18%, and the highest content of esters was found in JX, Liu, and LJ, which accounted for 50.90%, 30.57%, and 25.15%, respectively. The content of ester compounds in MJ samples was also relatively high, accounting for 22.77%.

The results in Figure 1c showed that the 11 volatile metabolites common to MBSL from the four different workshops were 1-Hexanol, 2-Nonen-4-one, 1-Iodododecane, 1-Iodotetracosane, Methyl stearate, 9-Octadecenoic acid(Z)-,methyl ester, Pentadecanal, Dimethyl ether, Disulfide dimethyl, Dimethyl trisulfide, and Dimethyl sulfone, which may be the key metabolites to distinguish the flavor of traditional MBSL from other foods. In Figure 1a, it can be seen that, except for Dimethyl trisulfide, the other 10 substances had relatively high contents, which may play an important role in the odor of MBSL. 1-Hexanol has a unique aroma of alcohols, and 1-Iodododecane, Iodotetracosane, as an iodinated hydrocarbon compound, may bring it subtle iodine or oceanic flavors. 9-Octadecenoic acid (Z)-methyl ester can bring a fresher or fruity flavor to food, with higher levels in all four samples; Aldehyde compounds usually have strong aromas, and Pentadecanal may impart an aldehyde or fat aroma. Dimethyl ether may give a subtle sweetness or fruity aroma to food. Disulfide dimethyl, Dimethyl trisulfide has a unique flavor of sulfur compounds, which may give food a sulfur flavor or the vegetables a flavor such as onions and garlic. Dimethyl sulfone provides a unique, slightly sweet aroma that may give food a special taste or flavor. The MBSL samples all had wonderful aromas, which may be related to these 11 volatile metabolites.

#### 3.2.2. Principal Component Analysis and Differential Metabolite Analysis of Volatiles

**Figure 2:**
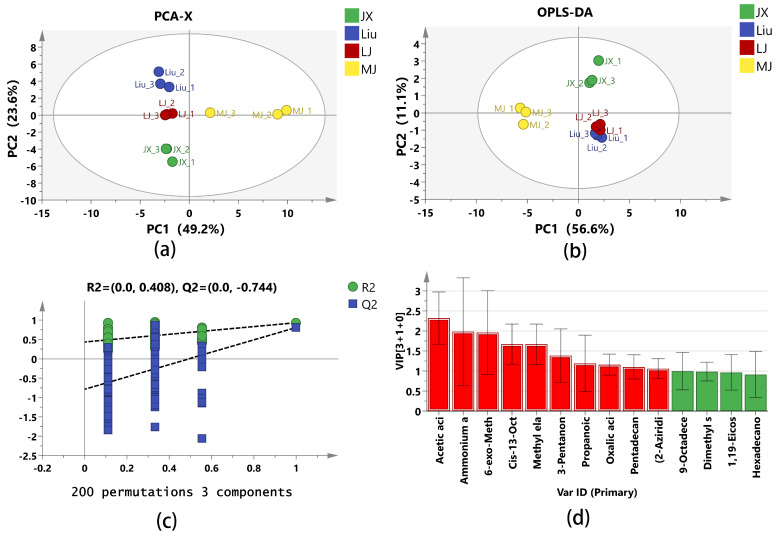
Principal component analysis and significant differential metabolite analysis of volatile compounds in MBSL. Notes: (**a**) PCA, (**b**) OPLS-DA model, (**c**) displacement test results, (**d**) VIP image.

The principal component analysis (PCA) was performed using SIMCA 14.1 software (Figure 2a). The first principal component (PC1) and the second principal component (PC2) contributed 49.2% and 23.6% of the variance, respectively, with a cumulative contribution of 72.8%, which indicated MBSL had distinct flavor characteristics, and that the MBSLs from the four different administrative districts had different flavor profiles. From the PCA, it can be seen that JX, Liu, and LJ samples have similarity in PC1 principal components, with significant differences from MJ. LJ and MJ are more similar in PC2 principal components, and are more obviously different from JX and Liu.

To investigate the differences in volatile metabolites in MBSL, we used SIMCA 14.1 software to build an OPLS-DA model (Figure 2b). The R^2^X = 0.92, R^2^Y = 0.97, and Q^2^ = 0.873 all exceeded 0.5, indicating good interpretation and prediction ability. A 200-time permutation test showed that R^2^ was greater than Q^2^, and the intercept of Q^2^ on the Y–axis was negative, confirming that the OPLS-DA model is reliable without overfitting (Figure 2c). Differential metabolites were selected based on variable importance in projection (VIP ≥ 1 and *p* < 0.05) (Figure 2d). A total of 10 differential volatile metabolites were screened, including Acetic acid, Oxalic acid, 6-exo-Methylbicyclo [2.2.1]hept-2-ene-5-endo-carboxylic acid, Cis-13-Octadecenoic acid, Methyl elaidate, 9-Octadecenoic acid(Z)-, Methyl ester, 3-Pentanone, Propanoic acid anhydride, Ammonium acetate, Pentadecanal, and (2-Aziridinylethyl)amine.

Acetic acid produces a distinct sour taste, Oxalic acid, as an organic acid, also has a slight sour taste. They were all detected in the MJ samples, and the content was much higher than in the other three samples, which may be the reason why the MJ sample smells the most acidic. In addition, only Acetic acid was tested in the LJ sample, and the content was not high. Acetic acid and Oxalic acid were not found in the JX and Liu samples, which may be the reason why the acidity of the LJ, JX, and Liu samples was lower than in the MJ one.

### 3.3. Non-Targeted Metabolomics Analysis of MBSL

#### 3.3.1. Principal Component Analysis of Metabolites

**Figure 3:**
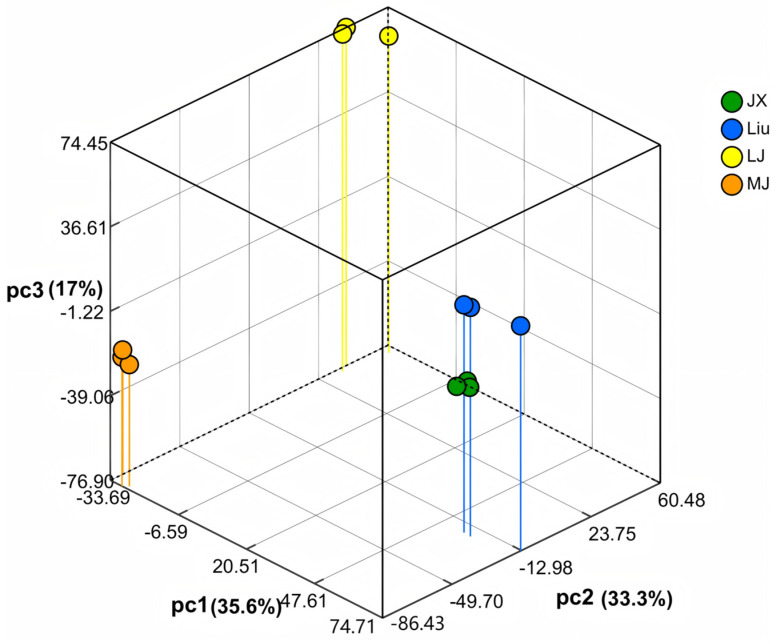
Principal component analysis (PCA) of non-volatile metabolites in MBSL.

To explore the similarities and differences of the metabolites in MBSL, the PCA analysis was performed, and the results are shown in Figure 3. The variable contributions of the first principal component PC1, the second principal component PC2, and the third principal component PC3 were 35.6%, 33.3%, and 17.0%, respectively. From the PCA, it can be seen that the samples within the same group were close to each other, indicating good repeatability and reliability. The clusters formed by the samples from the four workshops are widely dispersed, suggesting that there are significant differences in the overall non-volatile metabolites among the groups. This indicates that the metabolites in MBSLs from different workshops in Luoyang vary significantly. These differences are likely related to the distinct microbial fermentation systems.

#### 3.3.2. Classification of Metabolites Based on HMDB

In both ESI-positive and ESI-negative modes, a non-targeted metabolomics analysis of MBSL samples was conducted using the UHPLC-Q Exactive HF-X system (Thermo Fisher Scientific, Waltham, MA, USA). After data processing, 2622 metabolites were detected. A total of 1703 metabolites were identified according to the HMDB database.

**Figure 4:**
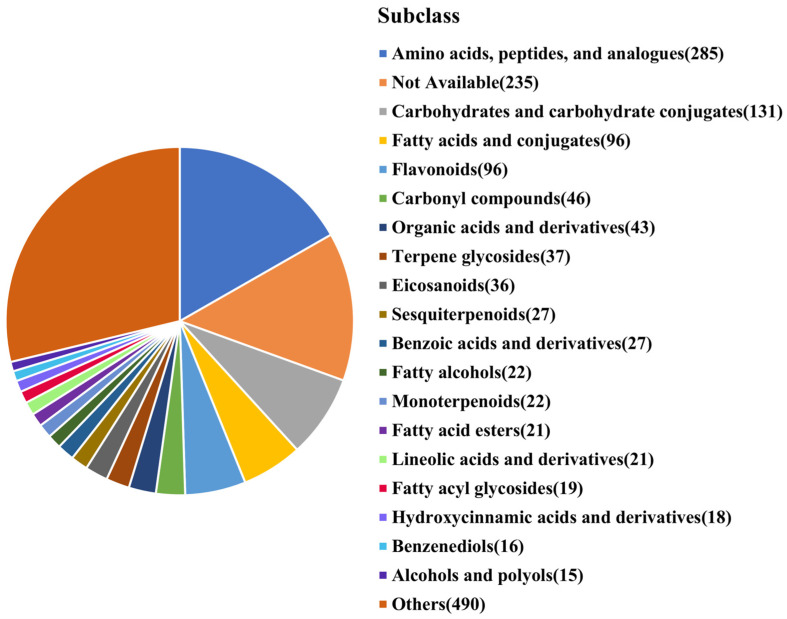
HMDB classification of non-volatile metabolites in MBSL.

As shown in Figure 4, the identified metabolites were classified into various subclasses according to HMDB information: amino acids, peptides, and analogues (285 types), carbohydrates and carbohydrate conjugates (131 types), fatty acids and conjugates (96 types), flavonoids (96 types), carbonyl compounds (46 types), and organic acids (43 types). These six categories accounted for 40.93% of the identified metabolites. Key metabolites in these categories include:

- Amino acids: glutamyl-s-methylcysteine, L-norleucine, L-leucine, gamma-glutamyl-s-methylcysteine sulfoxide, L-proline, L-valine, gamma-glutamylphenylalanine, L-phenylalanine, and L-theanine.
- Flavonoids: isovitexin, vitexin, 3′-hydroxy puerarin, astilbin, apigenin, apigenin 7-O-beta-D-rutinoside, and orientin.
- Organic acids: citric acid, L-lactic acid, glutaric acid, malic acid, succinic acid, isocitric acid, and L-tartaric acid.

These metabolites resulted from the microbial fermentation of mung bean’s primary nutrients, including proteins, starch, fats, and polyphenols, and they endow MBSL with nutritional value and many effects such as cooling, diuresis, cough relief, and immune enhancement etc. On the other hand, they also have a certain impact on the taste of MBSL.

#### 3.3.3. Differential Metabolites Analysis

**Figure 5:**
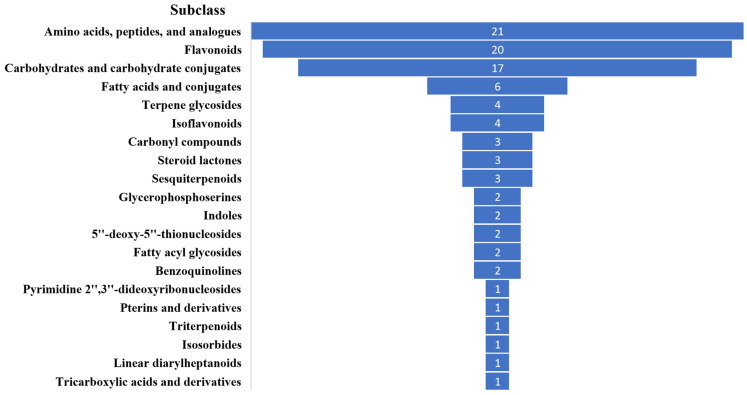
Classification information of significantly different non-volatile metabolites.

According to the VIP ≥ 1.0, *p* < 0.05 principle, there were 912 differential metabolites, indicating that the four samples, originating from different regions, have significant flavor differences. When the threshold was set to VIP ≥ 1.5, 189 significantly different metabolites were selected, mainly including: amino acids, peptides, and analogues (21 compounds), flavonoids (flavonoid glycosides, flavans, flavones, 20 compounds), carbohydrates and carbohydrate conjugates (17 compounds), fatty acids and conjugates (6 compounds), isoflavonoids (4 compounds), and organic acids (1 compound). The top 20 classification information in terms of quantity among 189 differential metabolites is shown in Figure 5, with the most abundant categories among the differential metabolites being amino acids, peptides, and flavonoids.

#### 3.3.4. KEGG Pathway Enrichment Analysis of Differential Metabolites

To clarify the fermentation metabolic mechanisms, a KEGG pathway enrichment topological analysis was performed on the 189 significantly differential metabolites. A total of 61 metabolic pathways were identified, with 12 showing significant differences (*p* < 0.05), as shown in Table 3. Using an impact score greater than 0.1 as the criterion, the pathways with the highest overall importance were flavonoid biosynthesis, flavonoid degradation, and tryptophan metabolism, which enriched 7, 5, and 3 differential metabolites, respectively. These metabolites were mainly: Eriodictyol, (−)-epigallocatechin, apigenin, (+)-taxifolin, Chrysin, Epigallocatechin, (+/−)-Catechin, Genistein, Phloroglucinol, 5-Hydroxykynurenamine, Serotonin, and L-Tryptophan. These metabolites are primarily involved in flavonoid biosynthesis and degradation as well as tryptophan metabolism, highlighting their significant roles in the fermentation process and the characteristic flavor development of MBSL.

Eriodictyol, (−)-Epigallocatechin, Apigenin, (+)-taxifolin, Chrysin, Epigallocatechin, and (+/−)-Catechin were found involved in flavonoid biosynthesis. These compounds are natural polyphenolic derived from plants, and they exhibit various pharmacological activities, including antioxidant, anti-inflammatory, and anticancer effects. In addition, the key metabolites including Eriodictyol, Genistein, Apigenin, (+)-taxifolin, and Phloroglucinol were involved in the flavonoid degradation pathway. Metabolites involved in tryptophan metabolism included 5-Hydroxykynurenine, serotonin, and L-Tryptophan. L-Tryptophan is one of the essential amino acids in humans. Through the aromatic amino acid metabolic pathway, it is converted into the neurotransmitter serotonin, which plays a role in regulating mood, sleep, and appetite control.

### 3.4. Microbial Analysis

#### 3.4.1. Alpha Diversity Analysis

To explore the microbial structural diversity in MBSL from different workshops, the bacterial and fungal community structures were preliminarily characterized using high-throughput sequencing. The Chao index estimates the number of OTUs in a sample, with a higher value indicating greater community richness. The Shannon index is used to estimate microbial diversity within a sample, with a higher value indicating greater community diversity.

As shown in Table 4, the coverage of sample sequences was over 99%, indicating that the vast majority of bacteria and fungi in the samples could be detected by this sequencing. The Chao index for fungi and bacteria in the MJ sample was significantly higher than that of the other three samples (*p* < 0.05), suggesting that the MJ sample has the richest microbial community.

The bacterial Shannon index for MJ, JX, and Liu was significantly higher than that of the LJ sample (*p* < 0.05), indicating that the bacterial diversity in these samples is greater than in LJ. Moreover, the Shannon index for MJ was significantly higher than the other three samples (*p* < 0.05), indicating that MJ has the highest fungal diversity, followed by LJ, Liu, and JX. Thus, the microbial composition in the MJ sample is the most diverse and rich.

#### 3.4.2. Bacterial Phylum and Genus-Level Community Composition Analysis

**Figure 6:**
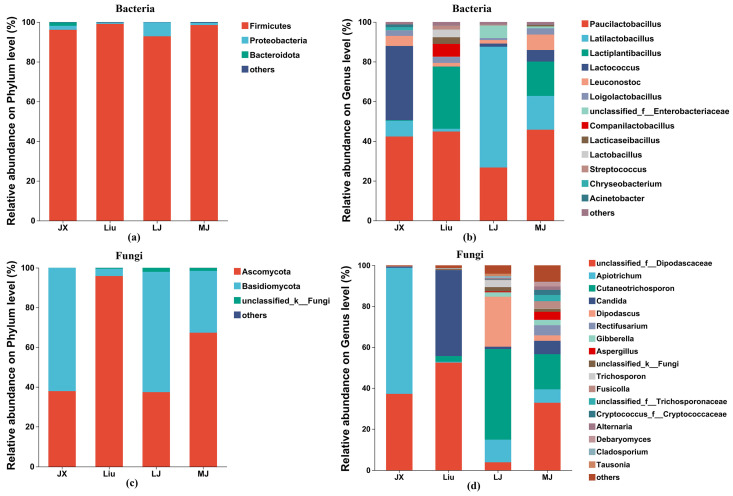
The relative abundance of the bacterial and fungal composition in MBSL from different workshops. Notes: species with relative abundance of less than 1% are included in others. (**a**): Bacteria-phylum level. (**b**): Bacteria-genus level. (**c**): Fungi-phylum level. (**d**): Fungi-genus level.

At the phylum level, a total of 12 bacterial species were identified in the MBSL samples, The main advantageous bacterial phyla were concluded to be *Firmicutes* (96.63%), *Proteobacteria* (2.66%), and *Bacteroidota* (0.51%), as shown in Figure 6a. It can be observed that *Firmicutes* had the highest average abundance in all samples, ranging from 92.83% to 99.90%. In the JX sample, the dominant bacterial phylum and its abundance was *Firmicutes* (96.11%), followed by *Proteobacteria* (2.01%) and *Bacteroidota* (1.74%). In the Liu sample, the dominant bacterial phylum was also *Firmicutes* (99.09%), with *Proteobacteria* and *Bacteroidota* having abundances of less than 1%. In the LJ sample, the dominant phylum was *Firmicutes* (92.83%), followed by *Proteobacteria* (7.01%), and *Bacteroidota* of less than 1%. In the MJ sample, the dominant phylum was *Firmicutes* (98.49%), followed by *Proteobacteria* (1.03%), and *Bacteroidota* of less than 1%.

At the genus level, a total of 106 bacterial genera were detected across all the samples, and bacterial genera with high abundance were screened, including *Paucilactobacillus* (average abundance 39.92%), *Latilactobacillus* (21.79%), *Lactiplantibacillus* (12.23%), *Lactococcus* (11.19%), *Leuconostoc* (4.13%), *Loigolactobacillus* (2.47%), *unclassified_f_Enterobacteriaceae* (1.87%), *Companilactobacillus* (1.62%), *Lacticaseibacillus* (1.04%), *Lactobacillus* (0.97%), *Streptococcus* (0.59%), *Chryseobacterium* (0.41%), and *Acinetobacter* (0.34%). As shown in Figure 6b, it can be seen that, in the JX sample, the dominant genera were *Paucilactobacillus* (42.33%), Lactococcus (37.37%), *Latilactobacillus* (7.95%), *Loigolactobacillus* (2.75%), and *Chryseobacterium* (1.56%), with the first two genera accounting for 79.7% of the total bacterial population. In the Liu sample, the dominant genera were *Paucilactobacillus* (44.84%) and *Lactiplantibacillus* (31.30%), accounting for 76.14% of the total bacterial population. In the LJ sample, the dominant genera were *Latilactobacillus* (60.81%) and *Paucilactobacillus* (26.72%), which together accounted for 87.53% of the total bacterial population. In the MJ sample, the dominant genera were *Paucilactobacillus* (45.77%), *Lactiplantibacillus* (17.31%), and *Latilactobacillus* (17.01%), making up 80.09% of the total bacterial population. *Paucilactobacillus* was the first dominant bacterial genus in JX, Liu, and MJ.

#### 3.4.3. Fungal Phylum and Genus-Level Community Composition Analysis

At the phylum level, five fungal phyla were identified in the MBSL samples. The dominant fungal phyla were mainly *Ascomycota* (59.59%), *Basidiomycota* (39.37%), and *unclassified_k_Fungi* (1.02%), which is consistent with previous reports [1]. As shown in Figure 6c, the relative abundances of *Ascomycota* in JX, Liu, LJ, and MJ were 37.88%, 95.83%, 37.37%, and 67.29%, respectively. The relative abundances of *Basidiomycota* in JX, Liu, LJ, and MJ were 62.08%, 3.68%, 60.59%, and 31.11%, respectively. In terms of the abundance of the dominant fungal phyla, JX and LJ were quite similar.

As shown in Figure 6d, 106 fungal genera were identified, with 17 main fungal genera. The genera were ranked by total abundance as follows: *unclassified_f_Dipodascaceae* (31.59%), *Apiotrichum* (19.88%), *Cutaneotrichosporon* (16.06%), *Candida* (12.56%), *Dipodascus* (6.79%), *Rectifusarium* (1.24%), *Gibberella* (1.21%), *Aspergillus* (1.11%), *unclassified_k_Fungi* (1.02%), and *Trichosporon* (0.98%), etc. In the JX sample, the dominant genera were *Apiotrichum* (61.62%) and *unclassified_f_Dipodascaceae* (37.17%). In the Liu sample, the dominant genera were *unclassified_f_Dipodascaceae* (52.47%), *Candida* (42.04%), and *Cutaneotrichosporon* (2.76%). In the LJ sample, the dominant genera were *Cutaneotrichosporon* (44.29%), *Dipodascus* (24.47%), and *Apiotrichum* (10.95%). In the MJ sample, the dominant genera were *unclassified_f_Dipodascaceae* (32.84%) and *Cutaneotrichosporon* (17.18%). It can be seen that the fungal community composition and abundance at the genus level differed significantly among the four MBSL samples from different administrative districts. These differences are also distinct from previous reports, which may be attributed to variations in the fungal strains used in the workshops or environmental factors.

### 3.5. Correlation Analysis Between Microbiome and Volatile Compounds

**Figure 7:**
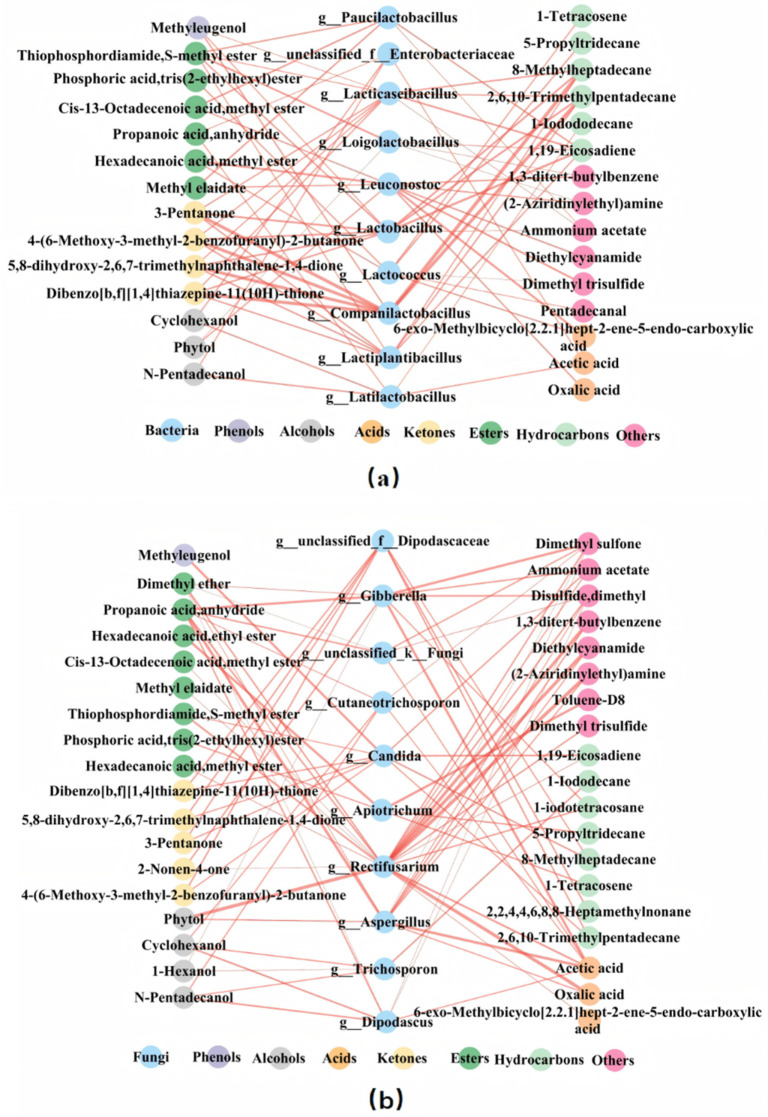
Positive correlation network between microbial genera and volatile metabolites of MBSL. Notes: (**a**) Bacteria, (**b**) fungi.

A correlation analysis was performed on the top 10 most abundant bacteria or fungi and 42 volatile metabolites. The selection criteria were r > 0.6 and *p* < 0.05, and the results are shown in Figure 7.

In Figure 7a, Methyleugenol (a compound with sweet and clove-like odors) was positively correlated with *Companilactobacillus*, *Lacticaseibacillus*, and *Lactobacillus*. Ketone compounds such as dibenzo[b,f][1,4]thiazepine-11(10H)-thione, 5,8-dihydroxy-2,6,7-trimethylnaphthalene-1,4-dione, 3-Pentanone, 4-(6-Methoxy-3-methyl-2-benzofuranyl)-2-butanone, and hydrocarbon compounds like 1,19-Eicosadiene, 2,6,10-Trimethylpentadecane, and 8-Methylheptadecane all showed significant positive correlations with *Lactiplantibacillus*, *Companilactobacillus*, *Lacticaseibacillus*, and *Lactobacillus*. Among alcohols, Cyclohexanol and N-Pentadecanol were significantly positively correlated with *Latilactobacillus* and *Enterobacteriaceae*. In esters, Hexadecanoic acid, Methyl ester showed a significant positive correlation with *Leuconostoc* and *Lactococcus*. Cis-13-Octadecenoic acid, methyl ester, and Methyl elaidate were significantly positively correlated with *Leuconostoc* and *Paucilactobacillus.* Phosphoric acid, tris (2-ethylhexyl) ester was significantly positively correlated with *Lactococcus*.

In Figure 7b, Methyleugenol is positively correlated only with *Candida*. Many ketone compounds along with hydrocarbon compounds like 2,6,10-Trimethylpentadecane and 8-Methylheptadecane, were all significantly positively correlated with *Dipodascaceae* and *Candida*. Cyclohexanol and N-Pentadecanol are positively correlated with *Cutaneotrichosporon*, *Dipodascus*, and *Trichosporon*. Phytol is significantly positively correlated with *Rectifusarium* and *Aspergillus*. Some esters such as Thiophosphordiamide, S-methyl ester, Cis-13-Octadecenoic acid, Methyl ester, Methyl elaidate, Hexadecanoic acid, methyl ester are positively correlated with *Rectifusarium*. Both Acetic acid and Oxalic acid are significantly positively correlated with *Rectifusarium* and *Aspergillus*.

Based on the number and the thickness of the connecting lines, it can be concluded that Methyleugenol is more strongly correlated with bacteria, suggesting that phenolic compounds may have a greater relationship with bacteria. Alcohols have more and thicker connections with fungi, indicating a stronger correlation with fungi. Similarly, Oxalic acid and Acetic acid likely have a greater relationship with fungi, while ketones are more closely related to bacterial genera. Esters show a close relationship with both fungal and bacterial genera, while Hydrocarbons are more closely associated with bacterial genera.

In addition, *Lactobacillus*, *Lacticaseibacillus*, *Companilactobacillus*, and *Lactiplantibacillus* were significantly correlated with 10, 10, 8, 8 volatile substances and were considered important bacteria. *Candida*, *Rectifusarium*, *Gibberella*, and *unclassified_f_Dipodascaceae* are significantly related with 10, 8, 7, 7 volatile compounds, respectively, and can be considered important fungi. These microbial genera maybe play a crucial role in the metabolism of volatile substances.

### 3.6. Correlation Analysis Between Microbiome and Non-Targeted Metabolomics

**Figure 8:**
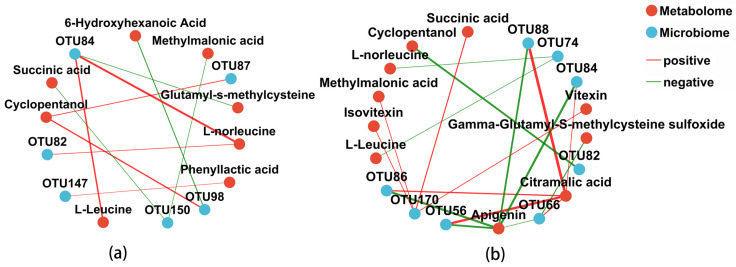
Correlation analysis of dominant microorganisms and some differential metabolites of MBSL. Notes: (**a**) Bacteria, (**b**) fungi.

A correlation analysis was conducted between the top 10 most abundant bacteria or fungi and 13 differential metabolites (including L-Leucine, L-norleucine, Glutamyl-s-methylcysteine, Cyclopentanol, 6-Hydroxyhexanoic Acid, Phenyllactic acid, Methylmalonic acid, Citramalic acid, Succinic acid, Apigenin, Isovitexin, and Vitexin). These compounds were selected from the top 50 metabolites. The selection criteria were |r| > 0.6 and *p* < 0.05, and the results are shown in Figure 8. Red lines indicate positive correlations, green lines indicate negative correlations, and the thickness of the lines corresponds to the absolute value of the correlation coefficient. The more connections a point has, the more important it is.

In Figure 8a, L-norleucine was significantly positively correlated with *Loigolactobacillus* (OTU84) and *Leuconostoc* (OTU82), with correlation coefficients of 0.88 and 0.64, respectively. L-leucine was also positively correlated with *Loigolactobacillus* (OTU84), with a correlation coefficient of 0.79. Cyclopentanol was strongly positively correlated with *Lactococcus* (OTU98 and OTU87), with correlation coefficients of 0.76 and 0.67, respectively. Other correlations are negative.

In Figure 8b, Citramalic acid was positively correlated with *Cutaneotrichosporon* (OTU88, OTU86 and OTU66), *Apiotrichum* (OTU56), and *Dipodascus* (OTU84), with correlation coefficients of 0.90, 0.85, 0.70, 0.67, and 0.63, respectively. Succinic acid, Isovitexin, Vitexin, and Methylmalonic acid were positively correlated with *Rectifusarium* (OTU170), with correlation coefficients of 0.67, 0.63, 0.63, and 0.63, respectively. Other metabolites showed significant negative correlations.

It can be observed that the differences in metabolites such as L-norleucine, L-leucine, and Cyclopentanol are strongly correlated with bacteria, primarily *Lactococcus*, *Loigolactobacillus*, and *Leuconostoc*. The metabolic differences in Citramalic acid, Succinic acid, Isovitexin, Vitexin, and Methylmalonic acid are mainly associated with fungi, with the dominant fungal genera being *Cutaneotrichosporon*, *Apiotrichum*, *Dipodascus*, and *Rectifusarium*. Therefore, amino acids and alcohol metabolites are more likely influenced by bacteria, while the metabolism of organic acids and flavonoid compounds may involve more fungal participation.

## 4. Discussion

Metabolomics analysis based on HS-SPME/GC-MS and LC-MS has been proven to be an effective and efficient method for detecting volatile and non-volatile flavor compounds in biological systems and fermented foods. For example, volatile and non-volatile flavor compounds in Northeast sauerkraut and jujube wine were detected by HS-SPME/GC-MS and LC-MS, providing a theoretical basis for the optimization of fermentation processes [11,12]. The high-throughput sequencing (HTS) technology of 16S rRNA can more accurately and quickly reveal the microbial community structure of samples. By revealing the correlation between microorganisms and metabolites, it is of great significance for optimizing fermentation processes, improving product quality, and developing functional fermented foods. Tang Jie et al. [10] detected flavor substances in fermented grains at different fermentation times based on HS-SPME-GC-MS, and conducted a correlation analysis with dominant microorganisms, and the results showed that esters and alcohols were related to the succession of lactobacillus and yeast communities, providing strategies for further improving the quality of Baijiu. Therefore, the multi-omics methods were used in MBSL, aiming to more accurately and clearly reveal the fermentation mechanism of MBSL.

In this study, 42 volatile compounds were detected, including 1 phenol, 4 alcohols, 3 acids, 5 ketones, 8 esters, 10 hydrocarbons, and 11 other compounds. Li Xuan et al. [1] used HS-SPME-GC-MS at 50 °C for extraction and detection, and found 18 alcohols, 6 acids, 5 phenols, and 3 esters in MBSL samples from three workshops. The results showed notable differences, with the same compounds including Methyleugenol, 1-Hexanol, Acetic Acid, Disulfide dimethyl, and Dimethyl trisulfide. And another 36 compounds were all different, among which the biggest difference was mainly reflected in esters and alcohols. We found more esters including Thiophordiamide, S-methyl ester, Phosphoric acid,tris(2-ethylhexyl) ester, Hexadecanoic acid,methyl ester, Hexadecanoic acid,ethyl ester, Methyl stearate, 9-Octadecenoic acid(Z)-,methyl ester, Cis-13-Octadecenoic acid,methyl ester, and Methyl elaidate, while three esters (Isopropyl palmate, 1,2-Benedicticarboxylic acid butyl octyl ester, and Methyl salicylate) were proposed in the report of Li Xua, and there was no intersection between the two. We found fewer alcohols—only four types (1-Hexanol,Cyclohexanol,Phytol,N-Pentadecanol—which are different from the other 18 alcohols (1-Hexanol, Ethanol, 2-Hexen-1-ol,(E)-, Benzyl alcohol, 3-Hexen-1-ol,(E)-, 2,3-Butanediol, 1-Nonanol, 2-Hexadecanol, 1-Heptanol, 3-Pentanol, 2-Penten-1-ol,(Z)-, Geraniol, 1-Hexadecanol,2-methyl-, 2-Hepten-1-ol,(Z)-, 3-Nonen-1-ol,(Z)-, Phenylethyl Alcohol, Methanethiol, 2-Butanol), and only 1-Hexanol is the same here. These differences are likely due to variations in extraction temperature and instrumental conditions. Previous studies have found that the aromatic compounds in raw ‘douzhi’ were significantly higher in variety and content compared to cooked ‘douzhi’, with alcohols showing the most significant changes, from 18 to 12 compounds [13]. This indicates that extraction temperature can influence the type and number of volatile metabolites detected. In this study, a higher extraction temperature of 80 °C was used, which extracted more esters. Additionally, differences in sampling season and fermentation environments may also have influenced the metabolic composition of the samples. For example, research on liquor fermentation showed that climate conditions during different brewing seasons affect the physicochemical properties of the liquor fermentation system, thus influencing the microbial community structure and metabolic activity [14,15,16].

To understand the regional differences in MBSL samples, non-targeted LC-MS metabolomics was also performed, identifying 1703 metabolites. The most abundant metabolites included amino acids, peptides, carbohydrates, fatty acids, flavonoids, and organic acids. The metabolic compositions of the four MBSL samples varied greatly, indicating that different operational conditions and environmental microbiomes have a significant impact on their quality [17,18].

α-Diversity analysis of the four samples revealed significant differences in bacterial and fungal diversity and abundance across different workshops. At the phylum level, the dominant bacterial phyla in all samples were *Firmicutes* (96.63%) and *Proteobacteria* (2.66%), consistent with previous studies on MBSL [1] and fermented foods such as liquor mash [9] and vinegar [19]. At the genus level, 13 dominant bacterial genera were identified, with a high abundance of *Paucilactobacillus* (39.92%), *Lactiplantibacillus* (12.23%), *Latilactobacillus* (21.79%), *Lactococcus* (11.19%), *Leuconostoc* (4.13%), *Loigolactobacillus* (2.47%), *Companilactobacillus* (1.62%), *Lacticaseibacillus* (1.04%), and *Lactobacillus* (0.97%), all of which are LAB, comprising 95.36% of the total bacterial abundance. The results were significantly different from previous studies in composition diversity. Li Xuan et al. [1] detected only four bacterial genera in MBSL samples, including *Lactobacillus* (71.78%), *Lactococcus* (15.28%), *Leuconostoc* (10.08%), and *Lelliottia* (1.44%), and LAB accounts for 97.14%. The reason may be due to different sources of MBSL samples.

The fungal community composition analysis showed that the dominant fungal phyla were *Ascomycota*, *Basidiomycota*, and *unclassified_k_Fungi*, consistent with previous studies, but with large differences in abundance [1]. At the genus level, 17 dominant fungal genera were identified, with *unclassified_f_Dipodascaceae* (31.59%), *Apiotrichum* (19.88%), *Cutaneotrichosporon* (16.06%), *Candida* (12.56%), *Dipodascus* (6.79%), and *Rectifusarium* (1.24%) being the most abundant. These genera are mostly yeasts, comprising 88.12% of the total fungal abundance. Li Xuan et al. [1] detected 11 dominant fungal genera, of which 8 genera were consistent with our findings, including *Dipodascus*, *Apiotrichum*, *Candida*, *unclassified_f_Trichosporonaceae*, *Alternaria*, *unclassified_k_Fungi*, *Cutaneotrichosporon*, and *Trichosporon*. Compared to bacteria, there were greater differences in the fungal genus composition across the different MBSL samples, with the MJ sample showing the highest fungal diversity, followed by LJ, Liu, and JX.

To investigate the relationship between volatile compounds, non-targeted metabolites, and the microbiome, correlation analyses were performed, while phenolic compounds such as Methyleugenol, ketones, and hydrocarbons are more closely related to bacterial genera, and some esters show a close relationship with both fungal and bacterial genera. The relationship between non-targeted metabolites and microbiomes showed amino acids and alcohol metabolites may be more likely influenced by bacteria, while the metabolism of organic acids and flavonoid compounds may involve more fungal participation. The microbial genera closely related to volatile metabolites were *Lactobacillus*, *Lacticaseibacillus*, *Companilactobacillus*, and *Lactiplantibacillus*, as well as the fungal genera *Candida*, *Rectifusarium*, *Gibberella*, and *unclassified_f_Dipodascaceae*. For non-target metabolites such as amino acids and flavonoids, the key microbial genera were *Lactococcus*, *Loigolactobacillus*, *Leuconostoc*, *Cutaneotrichosporon*, *Apiotrichum*, *Dipodascus*, and *Rectifusarium*. This was somewhat different from the conclusion in studies on douzhi that the fermentation was mainly caused by the bacteria *Leuconostoc* and *Lactococcus* [20].

## 5. Conclusions

The study analyzed the volatile metabolites, non-volatile metabolites, and microbial community composition in MBSL samples from four different workshops. The results showed:

(1) A total of 42 volatile compounds were detected in the samples, including 1 phenol, 4 alcohols, 3 acids, 5 ketones, 8 esters, 10 hydrocarbons, and 11 other compounds. The content of esters and acidic substances was relatively high, and 11 common volatile metabolites were identified across all samples, including 1-Hexanol, 2-Nonen-4-one, 1-Iodododecane, 1-Iodotetracosane, Methyl stearate, 9-Octadecenoic acid(Z)-,methyl ester, Pentadecanal, Dimethyl ether, Disulfide dimethyl, Dimethyl trisulfide, and Dimethyl sulfone.
(2) In total, 1703 metabolites were identified by non-targeted LC-MS, including amino acids, peptides, and analogues (285 types), carbohydrates and carbohydrate conjugates (131 types), fatty acids and conjugates (96 types), flavonoids (96 types), carbonyl compounds (46 types), and organic acids (43 types), etc.
(3) Volatile substances including phenolic compounds phenolic compounds such as Methyleugenol, ketones, and hydrocarbons are more closely related to bacterial genera, acidic substances (Acetic acid and Oxalic acid) in MBSL are more related to fungi, and some esters show a close relationship with both fungal and bacterial genera. Non-targeted metabolites including amino acids and alcohol metabolites may be more likely influenced by bacteria, and organic acids and flavonoid compounds may involve more fungal participation.
(4) The microbial genera closely related to volatile metabolites were *Lactobacillus*, *Lacticaseibacillus*, *Companilactobacillus*, and *Lactiplantibacillus*, as well as the fungal genera *Candida*, *Rectifusarium*, *Gibberella*, and *unclassified_f_Dipodascaceae*. For non-target metabolites such as amino acids and flavonoids, the key microbial genera were *Lactococcus*, *Loigolactobacillus*, *Leuconostoc*, *Cutaneotrichosporon*, *Apiotrichum*, *Dipodascus*, and *Rectifusarium.* These microorganisms may play an important role in the formation of the MBSL flavor.

This study can provide theoretical support for future research directions such as strain isolation, identification, strain enhancement, and further fermentation mechanisms exploration. At the same time, it also can lay a theoretical basis for stabilizing and improving the flavor quality of MBSL.

## Figures and Tables

**Table 1:** The geographical locations of the four samples.

Sample	Geographical Locations
JX	No. 2 Wuhan Road, Jianxi District, Luoyang, China
Liu	No. 86 Jiudu Middle Road, Xigong District, Luoyang, China
LJ	No. 6 Qianjin Street, Old Town District, Luoyang, China
MJ	No. 36 Dongxin’an Street, Chanhe Hui Ethnic District, Luoyang, China

**Table 2:** The sensory description of the four samples.

Sample	Sensory Description
JX	The texture was uniform but relatively thin, with a gray green color and a distinct green bean fragrance, with a light sour odor.
Liu	The texture was relatively uniform, slightly layered, with a greenish color, a light green bean aroma, and a slightly light sour odor.
LJ	The texture was uniform, with a grayish white green color, a light green bean aroma, and a slightly light sour odor.
MJ	The texture was uniform, with a grayish white green color and a distinct green bean aroma, with a strong sour odor.

**Table 3:** Pathway enrichment analysis of significantly different non-volatile substances.

Pathway Description	Pathway_ID	Num	Impact_Value	*p*_Value	*p*_Adjust
Flavonoid biosynthesis	map00941	7	0.11	0.00	0.00
Degradation of flavonoids	map00946	5	0.13	0.00	0.00
Galactose metabolism	map00052	3	0.02	0.01	0.12
Isoflavonoid biosynthesis	map00943	3	0.06	0.01	0.12
Aminoacyl-tRNA biosynthesis	map00970	3	0.00	0.01	0.10
Glycerophospholipid metabolism	map00564	4	0.09	0.01	0.10
Biosynthesis of various other secondary metabolites	map00997	3	0.07	0.01	0.09
Tryptophan metabolism	map00380	3	0.24	0.01	0.09
Thiamine metabolism	map00730	2	0.00	0.03	0.17
Phenylalanine, tyrosine and tryptophan biosynthesis	map00400	2	0.00	0.04	0.20
Biosynthesis of unsaturated fatty acids	map01040	2	0.00	0.04	0.20
Biosynthesis of cofactors	map01240	7	0.01	0.04	0.19

**Table 4:** Alpha diversity of bacteria and fungi in MBSL.

Sample	Bacteria			Fungi		
	Chao	Shannon	Coverage (%)	Chao	Shannon	Coverage (%)
JX	77.2 ± 8.82 b	1.68 ± 0.01 a	99.89 ± 0.04 a	23.33 ± 1.52 c	0.75 ± 0.02 d	100.00 ± 0 a
Liu	73.08 ± 8.45 b	1.76 ± 0.02 a	99.90 ± 0.03 a	68.51 ± 5.86 b	1.09 ± 0.02 c	100.00 ± 0 a
LJ	58.71 ± 10.07 b	1.15 ± 0.14 b	99.90 ± 0.02 a	51.83 ± 7.68 b	2.26 ± 00.25 b	100.00 ± 0 a
MJ	116.87 ± 14.93 a	1.71 ± 0.03 a	99.80 ± 0.03 a	94.44 ± 13.98 a	2.89 ± 0.09 a	100.00 ± 0 a

Note: Different letters in a column represent significant differences at the 0.05 level.

## Data Availability

The original contributions presented in this study are included in the article/Supplementary Material. Further inquiries can be directed to the corresponding authors.

## Supplementary Materials

The following supporting information can be downloaded at: www.mdpi.com/article/10.3390/foods14030511/s1, Table S1: Concentration content of volatile metabolites in MBSL from different workshops; OTUTaxonAnalysis_fungi; OTUTaxonAnalysis_bacteria.
