# Supplementary material for: Microbiome Flora and Metabolomics Analysis of Mung Bean Sour Liquid in Luoyang, China
Source: Foods. 2025 Feb 5;14(3):511. doi: 10.3390/foods14030511 (PMC11816372; doi:10.3390/foods14030511)
Supplement: Supplementary file 1 [file foods-14-00511-s001.zip › Supplementary Files/Table.1s.docx]

Table.1s. Concentration content of volatile metabolites in MBSL from different workshops

| Sequence | Metabolite | Content ± standard deviation (μg/L) | | | | Classification | MS | RI |
| --- | --- | --- | --- | --- | --- | --- | --- | --- |
|  |  | JX | Liu | LJ | MJ |  |  |  |
| 1 | Methyleugenol | / | 0.27±0.03 | 0.21±0.02 | 0.19±0.03 | Phenols | 178 | 2015.24 |
|  | Sum of percentage | 0 | 2.74% | 2.77% | 0.51% |  |  |  |
| 2 | Thiophosphordiamide,S-methyl ester | / | 0.13±0.02 | / | 0.49±0.13 | Ester | 126 | 1395.49 |
| 3 | Phosphoric acid,tris(2-ethylhexyl)ester | 0.16±0.02 | / | / | / | Ester | 71 | 1515.43 |
| 4 | Hexadecanoic acid,methyl ester | 0.21±0.05 | / | / | 0.29±0.13 | Ester | 74 | 2215.05 |
| 5 | Hexadecanoic acid,ethyl ester | 0.46±0.18 | / | 0.16±0.03 | / | Ester | 88 | 2251.75 |
| 6 | Methyl stearate | 0.57±0.09 | 0.41±0.05 | 0.63±0.04 | 0.74±0.27 | Ester | 74 | 2422.46 |
| 7 | 9-Octadecenoic acid(Z)-,methyl ester | 1.4±0.6 | 0.93±0.23 | 1.09±0.22 | 1.96±0.82 | Ester | 55 | 2440.74 |
| 8 | Cis-13-Octadecenoic acid,methyl ester | 1.42±0.59 | 0.79±0.3 | / | 2.43±0.97 | Ester | 83 | 2441.18 |
| 9 | Methyl elaidate | 1.42±0.59 | 0.79±0.3 | / | 2.43±0.97 | Ester | 69 | 2441.23 |
|  | Sum of percentage | 50.90% | 30.57% | 25.15% | 22.77% |  |  |  |
| 10 | 1-Hexanol | 0.17±0.01 | 0.54±0.02 | 0.55±0.04 | 0.64±0.23 | Alcohols | 56 | 1354.57 |
| 11 | Cyclohexanol | / | / | 0.09±0 | / | Alcohols | 57 | 1408.71 |
| 12 | Phytol | / | / | / | 0.55±0.21 | Alcohols | 57 | 2028.71 |
| 13 | N-Pentadecanol | / | / | 0.19±0.11 | / | Alcohols | 83 | 2275.47 |
|  | Sum of percentage | 1.51% | 5.42% | 11.01% | 3.27% |  |  |  |
| 14 | Acetic acid | / | / | 0.53±0.34 | 6.02±1.8 | Acids | 60 | 1481.12 |
| 15 | 6-exo-Methylbicyclo[2.2.1]hept-2-ene-5-endo-carboxylic acid | 1.8±0.36 | / | / | 3.53±2.07 | Acids | 66 | 1500.70 |
| 16 | Oxalic acid | / | / | / | 1.5±0.52 | Acids | 45 | 1225.62 |
|  | Sum of percentage | 16.27% | 0.00% | 7.10% | 30.18% |  |  |  |
| 17 | 2-Nonen-4-one | 0.28±0.03 | 0.34±0.02 | 0.31±0.11 | 0.75±0.32 | Ketones | 69 | 859.02 |
| 18 | 3-Pentanone | / | 0.71±0.14 | / | / | Ketones | 57 | 1312.98 |
| 19 | Dibenzo[b,f][1,4]thiazepine-11(10H)-thione | / | 0.07±0.04 | / | / | Ketones | 244 | 1485.22 |
| 20 | 5,8-dihydroxy-2,6,7-trimethylnaphthalene-1,4-dione | / | 0.28±0.11 | / | / | Ketones | 232 | 1981.84 |
| 21 | 4-(6-Methoxy-3-methyl-2-benzofuranyl)-2-butanone | / | 0.26±0.12 | / | / | Ketones | 232 | 1984.04 |
|  | Sum of percentage | 2.52% | 16.66% | 4.11% | 2.04% |  |  |  |
| 22 | 1-iodoicosane | 0.22±0.03 | 0.26±0.03 | 0.26±0.06 | / | Hydrocarbons | 71 | 1436.35 |
| 23 | 1-Iododecane | / | 0.07±0.08 | 0.21±0.06 | / | Hydrocarbons | 71 | 1599.98 |
| 24 | 5-Propyltridecane | 0.16±0.05 | / | / | / | Hydrocarbons | 43 | 1599.97 |
| 25 | 2,2,4,4,6,8,8-Heptamethylnonane | 0.14±0.01 | 0.14±0.02 | / | / | Hydrocarbons | 85 | 1627.27 |
| 26 | 1-iodotetracosane | 0.16±0.02 | 0.2±0.02 | 0.19±0.02 | 0.37±0.08 | Hydrocarbons | 71 | 1678.60 |
| 27 | 8-Methylheptadecane | / | 0.23±0.04 | / | / | Hydrocarbons | 71 | 1679.59 |
| 28 | 1-Iodododecane | 0.22±0.03 | 0.16±0.1 | 0.26±0.02 | 1.36±1.52 | Hydrocarbons | 71 | 1490.98 |
| 29 | 2,6,10-Trimethylpentadecane | / | 0.15±0.02 | / | / | Hydrocarbons | 71 | 1599.89 |
| 30 | 1,19-Eicosadiene | / | 0.37±0.01 | / | 0.8±0.41 | Hydrocarbons | 82 | 2029.03 |
| 31 | 1-Tetracosene | 0.23±0.09 | / | / | / | Hydrocarbons | 55 | 2275.19 |
|  | Sum of percentage | 10.08% | 15.86% | 12.24% | 6.89% |  |  |  |
| 32 | Disulfide,dimethyl | 0.35±0 | 0.4±0.11 | 0.45±0.04 | 1.23±0.38 | Others | 94 | 1080.39 |
| 33 | Dimethyl trisulfide | 0.16±0.02 | 0.11±0.01 | 0.09±0.03 | 0.38±0.07 | Others | 126 | 1393.44 |
| 34 | Dimethyl ether | 0.21±0.04 | 0.25±0.05 | 0.31±0.03 | 2.17±1.05 | Others | 45 | 951.32 |
| 35 | (2-Aziridinylethyl)amine | / | / | / | 1.26±0.44 | Others | 44 | 838.55 |
| 36 | Diethylcyanamide | / | / | / | 0.22±0.13 | Others | 70 | 1660.04 |
| 37 | 1,3-ditert-butylbenzene | / | / | / | 0.09±0.01 | Others | 175 | 1425.16 |
| 38 | Toluene-D8 | 0.12±0.02 | / | 0.07±0.01 | / | Others | 100 | 1823.54 |
| 39 | Propanoic acid,anhydride | / | / | 0.5±0.03 | 1.11±0.38 | Others | 57 | 1139.07 |
| 40 | Pentadecanal | 0.94±0.07 | 0.34±0.04 | 0.25±0.11 | 0.56±0.19 | Others | 82 | 2028.61 |
| 41 | Ammonium acetate | / | 1.37±1.09 | 0.71±0.22 | 4.14±2.45 | Others | 60 | 1503.58 |
| 42 | Dimethyl sulfone | 0.3±0.01 | 0.39±0.08 | 0.46±0.02 | 1.4±0.39 | Others | 94 | 1080.08 |

Note: **/** indicates undetected
